# A Multi-Faceted Evaluation of Impulsivity Traits and Early Maladaptive Schemas in Patients with Anorexia Nervosa

**DOI:** 10.3390/jcm10245895

**Published:** 2021-12-15

**Authors:** Paolo Meneguzzo, Patrizia Todisco, Enrico Collantoni, Valentina Meregalli, David Dal Brun, Elena Tenconi, Angela Favaro

**Affiliations:** 1 Department of Neuroscience, University of Padova, Via Giustiniani 2, 35128 Padova, Italy; enrico.collantoni@unipd.it (E.C.); valentina.meregalli@gmail.com (V.M.); elena.tenconi@unipd.it (E.T.); angela.favaro@unipd.it (A.F.); 2 Eating Disorders Unit, Casa di Cura “Villa Margherita”, 36057 Arcugnano, Italy; patrizia.todisco1964@gmail.com; 3 Padova Neuroscience Center, University of Padova, 35128 Padova, Italy; 4 Department of Linguistic and Literary Studies, University of Padova, 35128 Padova, Italy; daviddb@live.it

**Keywords:** impulsivity, anorexia nervosa, early maladaptive schema, schema domain, restraint, binge-purge

## Abstract

(1) Background: patients with Anorexia Nervosa (AN) are classified either as restrictive (ANr) or binge/purge (ANbp) according to the absence or presence of impulsive eating and compensatory behaviors. The aim of the present study was to assess the levels of impulsivity in both AN subtypes and to explore whether individual differences in impulsivity may be explained by differences in the presence of early maladaptive schemas. (2) Methods: the sample group included 122 patients with ANr, 112 patients with ANbp, and 131 healthy women (HW). All of these participants completed the UPPS-P scale for an assessment of impulsive behaviors and the Young Schema Questionnaire (YSQ-S3) for an assessment of early maladaptive schemas. (3) Results: the patients with ANbp displayed higher levels of impulsivity compared with the patients with ANr and HW. Patients with AN, especially the restrictive subtype, also reported higher levels of early maladaptive schemas than HW, and regression analyses revealed that specific maladaptive schemas partially explain the variability in impulsivity in both patients and HW. (4) Conclusions: it appears that maladaptive beliefs developed during childhood or adolescence may predict the development of impulsivity, a personality trait usually associated with maladaptive behaviors, and appears to be prevalent among ANbp patients. The clinical effects of this, as well as directions for future study, are also discussed in this paper.

## 1. Introduction

Anorexia Nervosa (AN) is a severe, life-threatening psychiatric disorder characterized by impaired body experience and perception. By means of dysfunctional eating behaviors, this disorder leads to self-starvation and an underweight condition that is linked to an intense fear of gaining weight, as well as to difficulty in emotional management [1,2,3]. From a clinical perspective, AN may be classified as restrictive (ANr) or binge eating/purging (ANbp) depending on the absence or presence of behaviors such as binge eating, purging, or other compensatory strategies such as excessive exercising or fasting [1]. This subtype distinction has been associated with specific clinical features, such as comorbidity and psychopathological characteristics [4,5,6,7], impulsivity, neurocognitive profiles, and treatment approaches [8,9,10]. Impulsivity has a role in the severity of clinical presentation, showing a predictive role in weight loss from the prodromic phase of the disorder [11,12]. Neurobiological correlates of impulsive behaviors have also been investigated, indicating the involvement of neuronal structures such as the insula and the reward system as well as the possible role of endocrinological factors [13,14,15,16]. However, further studies are needed for a more comprehensive evaluation of the differences in behaviors and psychopathology across the eating disorder spectrum and, specifically, in AN subtypes [17,18,19]. Indeed, the subtype classification has been criticized due to the high rate of diagnostic cross-over over time [20]. Some authors have suggested that various different latent variables, such as impulsivity or compulsivity, should be taken into consideration in order to better understand the classification [11]. This aspect is possibly linked to the presence of overlapping psychopathological elements, as has already been shown in bulimia nervosa [21]. The recent literature has focused specifically on differences in impulsivity traits between ED patients, showing the possible existence of a spectrum distribution from high self-control to high impulsivity traits [4,22].

Impulsivity is a personality trait that has been evaluated according to various domains (choice, motor, and trait impulsivity) and models [23]. One of the most widely-accepted theoretical approaches is a multidimensional model that addresses five different domains: positive and negative urgency, lack of perseverance, lack of premeditation, and sensation seeking (called the Urgency, Premeditation (lack of), Perseverance (lack of), Sensation Seeking, Positive Urgency, Impulsive Behavior Scale-UPPS-P) [24]. Positive urgency describes the propensity to act impulsively when feeling positive emotions, whereas negative urgency reflects the tendency to act impulsively when experiencing negative affects; lack of perseverance refers to the tendency to not persist in an activity that is boring, and lack of premeditation denotes the tendency to act without considering the consequences of one’s behavior, while sensation seeking indicates one’s disposition regarding seeking new and exciting experiences [25]. Impulsivity is considered a transdiagnostic trait that characterizes a wide range of maladaptive behaviors and psychopathological symptoms [26] and is linked to the severity of many psychiatric diseases such as personality disorders, substance use disorders, bipolar disorder, and eating disorders [27]. A growing body of research has found transdiagnostic associations between impulsivity traits and early childhood maltreatment, even if the underlying neurobiological and cognitive mechanisms are still unclear [28,29,30,31].

From a cognitive perspective, early maladaptive schemas (EMSs) are pervasive, self-defeating, and dysfunctional cognitive patterns of memories, emotions, and physical sensations that represent the lenses through which one sees the world [32]. According to Young’s definition, EMSs are developed during childhood or adolescence from universal psychological needs and from the interaction of temperament facets with early adverse relational experiences [33]. Early maladaptive schemas are linked to several psychopathologies and have been widely evaluated in the literature [34]. In eating disorders, EMSs have been linked to a more severe clinical presentation [35] as well as to dysfunctional responses to interpersonal scenarios [36]. Moreover, EMSs have been shown to play a mediation role between early maltreatment and impulsive behaviors, from binge eating to self-harm [37,38], which highlights the role that cognitive functioning may play in impulsivity [29]. An integrated cognitive model has been recently proposed as a possible explanation for the complex interaction of maladaptive schemas, emotional processing, and behavioral dysregulation, showing the effects of cognitive schemas on impulsivity as a consequence of dysfunctional emotional management [39]. From this perspective, behavioral dysregulation is the result of emotion-regulation ineffectiveness, which arises from schemas associated with disruptions in emotional engagement and experiences developed during childhood and adolescence. This theoretical model is based on several studies that have linked EMSs to emotional dysregulation in different psychiatric conditions characterized by impulsive maladaptive behaviors [40,41]. However, despite the high levels of EMSs in patients with AN and the presence of an impaired emotional regulation [2,42], no study has evaluated their role in the impulsivity facets in this specific population.

The current study aims to evaluate the predictive role of EMSs in impulsivity, both in AN subtypes and in the general population, examining for the first time specific features that could elucidate cognitive differences between restrictive and binge/purge behaviors in patients with EDs and that could be evaluated as possible therapeutic targets in future clinical trials. In accordance with the existing literature, our main aim is to find specific relationships between impulsivity traits and specific early maladaptive schemas, with a differentiation between clinical subgroups and the general population.

## 2. Materials and Methods

### 2.1. Participants

For this study, we enrolled consecutive patients at the Eating Disorders Center of the University Hospital of Padova and at the Eating Disorders Unit of the Casa di Cura “Villa Margherita” (in Arcugnano, Vicenza, Veneto, Italy). A comparison group from the general population was also enrolled through public advertisements. Inclusion criteria for the participants were as follows: (a) sufficient proficiency in the Italian language, (b) between 15 and 60 years of age, (c) self-attribution as a cisgender woman, and (d) no present or past psychotic or medical comorbidities. For the patients, we used an additional inclusion criterion: (e) having a full AN diagnosis based on DSM-5 [1]. For comparison, a convenient sample of healthy women (HW) were enrolled, and we added an exclusion criterion to this group: present or past diagnosis of any eating disorder (ED), both for themselves or first-degree relatives. We categorized patients in the restrictive (ANr) or binge-purge (ANbp) subtypes depending on the presence or absence of overeating episodes or purging behaviors for weight control. The final sample comprised 122 ANr patients, 112 ANbp patients, and 131 controls.

All participants (or their parents, if they were underage, i.e., <18 years old) signed an informed consent pertaining to the use of their clinical and psychological data. The present study was conducted in accordance with the latest version of the Declaration of Helsinki and approved by local ethics committees.

### 2.2. Materials and Procedure

Demographic data such as age, duration of the eating disorder, weight, and height were collected via a structured interview conducted by a trained researcher. During the interview, the inclusion and exclusion criteria were also assessed. All participants completed a series of self-reported questionnaires.

The eating disorder examination questionnaire (EDE-Q) is a widely used 28-item self-report questionnaire designed to evaluate psychopathological EDs, and it consists of four subscales—restraint, eating concerns, shape concerns, weight concerns—as well as a global score [43]. The items are scored on a 7-point Likert-type scale from 0 to 6, with higher scores indicating a higher degree of specific psychopathology. In this study, Cronbach’s α = 0.89.

The UPPS-P scale is a self-reported 20-item questionnaire that is used to assess five different domains: negative urgency (NU), positive urgency (PU), lack of perseverance (LPers), lack of premeditation (LPrem), and sensation seeking (SS) [44]. The items are scored on a 4-point Likert-type scale, from 1 to 4, with higher scores indicating higher impulsivity traits. This scale has been proven to offer a good degree of reliability and predictive validity in the Italian population [45,46]. In this study, Cronbach’s α = 0.85.

We evaluated early maladaptive schemas via the short version of the Young Schema Questionnaire (YSQ-S3), a self-reported 90-item tool used for assessing four domains: disconnection and rejection (DR), impaired autonomy and performance (IA), excessive responsibility and standards (ER), and impaired limits (IL) [47,48]. The items are scored on a 6-point Likert-type scale, from 1 to 6, with higher scores indicating higher presence of maladaptive schemas. In this study, Cronbach’s α = 0.95.

### 2.3. Statistical Analysis

We evaluated the demographic and psychological characteristics of the subgroups using different ANOVAs, with post hoc analyses corrected via the Bonferroni method. The data were verified for ANOVA assumptions—normal distribution, homogeneity of variances, and independence of the samples. Different hierarchical linear regressions were used to evaluate the relationship between the impulsivity profile and the maladaptive schema domains, using UPPS-P subscales as the dependent variable and EMS domains, age, and diagnosis as independent variables. Different regression models were evaluated for patients with AN and HW. We included age as the first regressor, as suggested by the developmental literature [49]; then, we included AN subtypes, as indicated by clinical feature; and finally, we included the EMS domains, looking for their specific role in impulsive traits. We estimated the effect size for the ANOVA analyses through the partial η^2^ (η^2^) coefficient (η^2^ = 0.01 was considered a small effect size, η^2^ = 0.06 was considered a medium effect, and η^2^ = 0.14 was considered a large effect). Differences were considered significant if *p* < 0.05. Post hoc power analysis showed a power (1 − β) = 0.99 with an α = 0.05 and a |ρ| = 0.3 (medium). The data were analyzed using SPSS Statistics Version 25.0 (IBM Corp, Armonk, NY, USA).

## 3. Results

Table 1 includes a description of the sociodemographic characteristics of the participants. Significant differences were found only with respect to BMI, as was expected. No differences emerged between the centers regarding the demographic characteristics of the participants.

**Figure 1:**
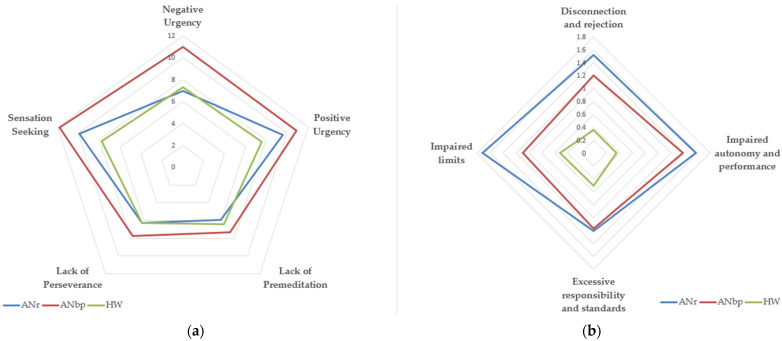
Graphical representation of the distribution of the UPPS-P (**a**) and YSQ-3 (**b**) subscales across the included samples. The graphs show the overlap between the subgroups as well as the differences in the mean distribution.

As regards psychopathology and impulsivity scores, we found significant differences between the two AN subgroups as well as between the patients and the controls. A graphical representation of the distributions is reported in Figure 1. In comparison with the other groups, ANbp patients reported higher scores than the other AN subtypes in all the UPPS-P subscales, and ANr patients showed a higher score in the EMS domains. See Table 2 for details.

The hierarchical regression analyses showed different relationships between age, diagnosis, EMS domains, and impulsivity, showing that subtype—rather than age—plays a role in the explanation of the impulsive behaviors in patients. Moreover, specific effects of the EMS domains in specific impulsive traits emerged in the regression analyses. See Table 3 and Table 4 for details.

Looking at the HW group, no regression model was significant only for age as regressor, but we did find that some specific EMS domains predicted specific impulsive traits. Furthermore, when comparing the regression models, specific relationships were found in the AN sample between negative urgency and disconnection and rejection; between lack of premeditation and impaired autonomy and performance; as well as between sensation seeking, impaired limits, and excessive responsibility and standards.

## 4. Discussion

This study corroborates the hypothesis that there is a significantly different impulsive profile in the two AN subtypes, and it highlights differences among healthy women that should be further analyzed. In line with our primary goal, we show specific EMS domains that have a role in impulsive traits, pointing out possible treatment targets for future studies.

As for impulsivity, our results show that patients with AN present clear differences between the two clinical subtypes, supporting the idea that a symptomatologic spectrum approach to AN diagnosis could improve clinical methods [4,13,50]. Indeed, patients with ANr showed specific differences when compared with healthy women only in sensation seeking and positive urgency scores. Patients with ANbp, on the other hand, showed significantly higher scores in all the impulsivity subdomains when compared with healthy peers. Impulsivity traits should be evaluated with the goal of a more detailed comprehension of AN psychopathology [50] because of its role in both clinical presentation and treatment outcome [51,52]. Differences in specific impulsivity traits between restrictive and binge/purge subtypes have already been found in patients with AN [22]. However, little is known about their relationship with cognitive schemas. In line with the findings of other studies, our results corroborate the presence of a top-down control in ANr patients [53,54], thereby showing that low levels of impulsivity are linked to negative emotions as well as a lack of premeditation or perseveration, which are more characteristic of cognitive rumination. This cognitive profile seems to reflect a tendency towards compulsive thoughts derived from the perpetuation of rewarding reinforcement linked to starvation [55]. However, restrictive patients reported a unique profile linked to impulsive behaviors due to positive emotions and sensational experiences, indicating that the specific role of positive affect in the ANr subtype [56] may not be controlled by the top-down rumination that characterizes these people and be expressed with impulsive facets. Therefore, our data seem to suggest the possible presence of a partial overlap between compulsive and impulsive facets in patients with AN. More evidence is needed to better understand the role of top-down cognitive control in AN, including the analysis of the emotional value of the stimuli used to evaluate impulsivity and cognitive rumination [57]. Finally, recent neurobiological evidence of the impairment in dopaminergic pathways in patients with AN might partially explain the overlap between compulsive and impulsive behaviors, thereby integrating neurocognitive aspects with biological markers [58].

As for the specific differences between patients with AN and healthy women, we found that the negative urgency trait is predicted by disconnection and rejection domains, a group of schemas that are highly consistent with patients with AN and include factors such as feeling defective, unwanted, and/or invalid in significant aspects as well as the expectation of being rejected and isolated by others [11,36]. These negative self-evaluations may be part of the negative self-judgment that has also been noted as a specific aspect in body image evaluation [59] as well as in the negative management of emotive responses [2,39]. For the lack of premeditation impulsive trait, we found in patients a negative association with impaired autonomy, a schema domain that describes concerns about the inability to manage difficulties without others’ help. This result is in line with the interpersonal difficulties of patients with AN and with self-inefficacy elements that have been pointed out as precipitant and maintenance factors [60]. It could also be linked to the cognitive overcontrol that has been described in patients with AN [4]. Finally, in patients with AN, the sensation seeking trait displayed a specific positive association with impaired limits and a negative association with excessive responsibility, indicating that impaired impulsive behaviors are correlated with seeking out novel and thrilling experiences, with difficulties in the identification of internal and external limits [61].

From a clinical perspective, our results add to the knowledge about impulsivity currently available in the field of AN, corroborating the idea that it is not simply a disorder of compulsivity [62]. More laboratory and clinical studies are needed in order to analyze different facets of impulsivity and their responsiveness to treatment approaches, as shown in other eating disorders [63,64]. To this end, maladaptive schemas might be pointed out as a target in treatment pathways that aim to evaluate the possible effects on impulsivity traits. Specific approaches are available from schema therapy, and early evaluation and treatment of EMSs might improve psychological distress in patients [65]. Indeed, both high levels of EMSs and high levels of impulsivity have negative effects on the outcome of eating disorders [10,35], and new possible targets for the implementation of current treatments are needed. Recent advantages in AN comprehension have underlined the need for personalized treatments [66], and impulsivity traits and maladaptive schemas should be included in patients’ profiles.

Our study has some strong points that should be highlighted. We recruited patients from different settings across the severity spectrum of AN, and we also applied well-validated questionnaires that obtained robust data. However, the present results should be considered to have several limitations. Firstly, for the evaluation of impulsivity traits, we only applied a self-report questionnaire, and future investigation should also use behavioral tasks. Secondly, in an attempt to provide more robust results, we included only women, which nevertheless leads to less generalizable evidence. Thirdly, this is a cross-sectional study and, for this reason, no cause–effect relationship (but only dependency of constructs) has been tested by means of regression analyses. Finally, the clinical sample also consisted of patients with other possible psychiatric co-morbidities, which might have influenced the results (i.e., anxiety disorder or personality traits) that have not been evaluated.

## 5. Conclusions

Impulsivity traits in AN are complex and understudied features that have several implications for their treatment and outcome. In this study, we aimed to evaluate the relationship between EMSs and impulsive traits, showing the relationships between specific cognitive schemas and impulsive behaviors. Different maladaptive schemas have shown the possible role of impulsive facets and a connection with ED psychopathological core elements. Our data highlighted specific links between cognitive schemas and particular facets of impulsivity, showing that the negative urgency, sensation seeking, and lack of premeditation traits have a specific role in the AN spectrum. Moreover, future studies should investigate the impact of specific psychological treatments on the relationship between impulsive traits and EMSs.

## Figures and Tables

**Table 1:** Demographic description of the sample.

	ANr *n* = 122	ANbp *n* = 112	HW *n* = 131	F	*p* η^2^
Age, years	23.89 (9.77) (22.13–25.64)	24.97 (7.98) (23.48–26.47)	23.88 (7.29) (23.49–24.26)	0.870	0.420 0.005
BMI, kg/m^2^	15.31 (1.79) (14.99–15.63)	16.54 (1.66) (16.24–16.85)	21.88 (3.31) (21.31–22.46)	267.501	<0.001 0.593
Duration of the disorder, years	4.51 (3.74) (4.00–5.02)	5.41 (3.88) (4.86–5.96)	-	1.590	0.210 0.010
Education, years	13.57 (2.55) (11.75–14.15)	14.24 (2.50) (13.93–15.40)	14.28 (3.11) (13.83–14.60)	2.217	0.111 0.041

The table reports means, standard deviations, and 95% confidence intervals. ANr: anorexia nervosa restricting; ANbp: anorexia nervosa binge-purging; HW: healthy women.

**Table 2:** Psychological evaluation of the sample.

	ANr	ANbp	HW	F	*p* η^2^	Post-Hoc
EDE-Q						
Restraint	4.76 (4.50) (3.95–5.57)	5.87 (4.84) (4.97–6.76)	1.91 (2.64) (1.51–2.31)	25.505	<0.001 0.009	ANr > HW (<0.001) ANbp > HW (<0.001)
Eating concerns	4.51 (4.19) (3.75–5.27)	5.96 (4.98) (5.04–6.88)	0.52 (1.03) (0.35–0.72)	55.634	<0.001 0.089	ANr > HW (<0.001) Anbp > HW (<0.001) Anbp > Anr (0.014)
Shape concerns	8.38 (9.51) (6.66–10.10)	11.03 (13.05) (8.62–13.44)	5.03 (5.00) (4.10–5.94)	9.883	<0.001 0.035	ANr > HW (0.038) ANbp > HW (<0.001)
Weight concerns	5.73 (4.95) (4.84–6.62)	6.55 (4.58) (5.34–7.77)	3.07 (2.90) (2.55–3.61)	13.463	<0.001 0.006	ANr > HW (<0.001) ANbp > HW (<0.001)
Global	5.85 (5.46) (4.86–6.83)	7.35 (7.09) (6.04–8.66)	2.63 (2.50) (2.17–3.15)	20.835	<0.001 0.011	ANr > HW (<0.001) ANbp > HW (<0.001)
UPPS-P						
NU	6.93 (4.40) (6.15–7.72)	10.99 (2.62) (10.52–11.46)	7.27 (2.16) (6.90–7.65)	60.916	<0.001 0.247	ANbp > HW (<0.001) ANbp > ANr (<0.001)
PU	9.57 (55.43) (8.60–10.54)	10.88 (1.89) (10.54–11.22)	7.59 (2.10) (7.22–7.95)	27.999	<0.001 0.131	ANbp > HW (<0.001) ANbp > ANr (0.012) ANr > HW (<0.001)
LPrem	5.90 (3.53) (5.27–6.53)	7.36 (2.85) (6.84–7.87)	6.39 (1.50) (6.13–6.65)	8.908	<0.001 0.046	ANbp > HW (0.016) ANbp > ANr (<0.001)
LPers	6.28 (4.22) (5.52–7.03)	7.75 (3.43) (7.13–8.37)	6.29 (1.91) (5.96–6.62)	8.090	<0.001 0.042	ANbp > HW (0.002) ANbp > ANr (0.001)
SS	9.90 (5.98) (8.84–10.97)	11.80 (2.45) (11.36–12.24)	7.78 (2.36) (7.37–8.19)	32.719	<0.001 0.150	ANr > HW (<0.001) ANbp > HW (<0.001) ANbp > ANr (0.001)
EMS						
Disconnection and rejection	1.52 (1.07) (1.33–1.71)	1.20 (1.17) (0.99–1.41)	0.35 (0.40) (0.29–0.42)	53.353	<0.001 0.223	ANr > HW (<0.001) ANbp > HW (<0.001) ANr > ANbp (0.023)
Impaired autonomy and performance	1.58 (0.95) (1.41–1.75)	1.38 (1.17) (1.17–1.59)	0.35 (0.39) (0.28–0.42)	69.658	<0.001 0.272	ANr > HW (<0.001) ANbp > HW (<0.001)
Excessive responsibility and standards	1.20 (0.82) (1.06–1.35)	1.17 (1.13) (0.96–1.37)	0.50 (0.42) (0.43–0.58)	28.460	<0.001 0.133	ANr > HW (<0.001) ANbp > HW (<0.001)
Impaired limits	1.72 (1.10) (1.52–1.92)	1.09 (1.22) (0.87–1.32)	0.52 (0.53) (0.42–0.61)	46.860	<0.001 0.207	ANr > HW (<0.001) ANbp > HW (<0.001) ANr > ANbp (<0.001)

The table reports means, standard deviations, and 95% confidence intervals. Post hoc analysis applied Bonferroni correction. ANr: anorexia nervosa restricting; ANbp: anorexia nervosa binge-purging; HW: healthy women; NU: negative urgency; PU: positive urgency: LPrem: lack of premeditation; LPers: lack of perseveration; SS: sensation seeking.

**Table 3:** Regression models for patients with AN.

		NU		PU		LPrem		LPers		SS	
Model/DV		Δ*R*^2^ *p*	*B* *p*	Δ*R*^2^ *p*	*B* *p*	Δ*R*^2^ *p*	*B* *p*	Δ*R*^2^ *p*	*B* *p*	Δ*R*^2^ *p*	*B* *p*
1	Age	0.018 0.044	**−0.136** **0.044**	<0.001 0.917	−0.007 0.917	0.027 0.016	**0.163** **0.016**	0.017 0.056	0.129 0.056	0.018 0.048	**0.133** **0.048**
2	Age	0.212 <0.001	**−0.176** **0.004**	0.028 0.047	−0.022 0.748	0.045 <0.001	**0.144** **0.029**	0.043 0.001	0.111 0.096	0.039 0.002	0.116 0.081
	Diagnosis		**0.463** **<0.001**		**0.167** **0.014**		**0.214** **0.001**		**0.209** **0.002**		**0.199** **<0.001**
3	Age	0.167 <0.001	**−0.213** **<0.001**	0.176 <0.001	−0.068 0.279	0.221 <0.001	0.087 0.143	0.175 <0.001	0.062 0.316	0.151 <0.001	0.067 0.286
	Diagnosis		**0.467** **<0.001**		**0.217** **0.001**		**0.380** **<0.001**		**0.309** **<0.001**		**0.209** **0.002**
	DR		**0.432** **0.005**		**0.567** **0.001**		**0.955** **<0.001**		**0.699** **<0.001**		**0.527** **0.003**
	IA		−0.301 0.073		−0.081 0.673		**−0.696** **<0.001**		−0.150 0.427		0.076 0.691
	ER		**−0.392** **<0.001**		**−0.544** **<0.001**		**−0.518** **<0.001**		**−0.352** **<0.001**		**−0.398** **<0.001**
	IL		**−0.102** **0.381**		−0.099 0.460		**0.404** **0.002**		0.040 0.762		**0.319** **0.017**

Independent variables: NU: negative urgency; PU: positive urgency: LPrem: lack of premeditation; Lpers: lack of perseveration; SS: sensation seeking. DV: dependent variables, DR: disconnection and rejection; IA: impaired autonomy and performance; ER: excessive responsibility and standards; IL: impaired limits. The significant results are reported in bold characters.

**Table 4:** Regression models for HW.

		NU		PU		LPrem		LPers		SS	
Model/Variables		Δ*R*^2^ *p*	*B* *p*	Δ*R*^2^ *p*	*B* *p*	Δ*R*^2^ *p*	*B* *p*	Δ*R*^2^ *p*	*B* *p*	Δ*R*^2^ *p*	*B* *p*
1	Age	<0.001 0.948	−0.006 0.948	0.001 0.701	−0.034 0.701	0.001 0.738	−0.030 0.738	0.004 0.481	−0.062 0.481	0.007 0.355	−0.081 0.355
2	Age	0.217 <0.001	−0.001 0.890	0.230 <0.001	−0.019 0.810	0.283 <0.001	−0.018 0.815	0.220 <0.001	−0.045 0.578	0.419 <0.001	−0.051 0.463
	DR		0.070 0.651		**0.600** **<0.001**		**0.642** **<0.001**		**0.712** **<0.001**		**0.333** **0.015**
	IA		0.403 0.058		−0.079 0.706		0.068 0.739		−0.104 0.626		0.196 0.288
	ER		**−0.233** **0.024**		**−0.263** **0.010**		**−0.403** **<0.001**		**−0.325** **0.002**		−0.054 0.548
	IL		0.114 0.490		0.121 0.459		**−0.490** **0.003**		−0.146 0.383		0.204 0.158

HW: healthy women; NU: negative urgency; PU: positive urgency: LPrem: lack of premeditation; LPers: lack of perseveration; SS: sensation seeking; DR: disconnection and rejection; IA: impaired autonomy and performance; ER: excessive responsibility and standards; IL: impaired limits. Significant results are reported in bold characters.

## Data Availability

The datasets used and analyzed during the current study are available from the corresponding author upon reasonable request.
